# High-efficiency transfection of cultured primary motor neurons to study protein localization, trafficking, and function

**DOI:** 10.1186/1750-1326-5-17

**Published:** 2010-04-21

**Authors:** Claudia Fallini, Gary J Bassell, Wilfried Rossoll

**Affiliations:** 1 Department of Cell Biology, Emory University School of Medicine, Atlanta 30322, USA; 2 Department of Neurology and Center for Neurodegenerative Diseases, Emory University School of Medicine, Atlanta 30322, USA

## Abstract

**Background:**

Cultured spinal motor neurons are a valuable tool to study basic mechanisms of development, axon growth and pathfinding, and, importantly, to analyze the pathomechanisms underlying motor neuron diseases. However, the application of this cell culture model is limited by the lack of efficient gene transfer techniques which are available for other neurons. To address this problem, we have established magnetofection as a novel method for the simple and efficient transfection of mouse embryonic motor neurons. This technique allows for the study of the effects of gene expression and silencing on the development and survival of motor neurons.

**Results:**

We found that magnetofection, a novel transfection technology based on the delivery of DNA-coated magnetic nanobeads, can be used to transfect primary motor neurons. Therefore, in order to use this method as a new tool for studying the localization and transport of axonal proteins, we optimized conditions and determined parameters for efficient transfection rates of >45% while minimizing toxic effects on survival and morphology. To demonstrate the potential of this method, we have used transfection with plasmids encoding fluorescent fusion-proteins to show for the first time that the spinal muscular atrophy-disease protein Smn is actively transported along axons of live primary motor neurons, supporting an axon-specific role for Smn that is different from its canonical function in mRNA splicing. We were also able to show the suitability of magnetofection for gene knockdown with shRNA-based constructs by significantly reducing Smn levels in both cell bodies and axons, opening new opportunities for the study of the function of axonal proteins in motor neurons.

**Conclusions:**

In this study we have established an optimized magnetofection protocol as a novel transfection method for primary motor neurons that is simple, efficient and non-toxic. We anticipate that this novel approach will have a broad applicability in the study of motor neuron development, axonal trafficking, and molecular mechanisms of motor neuron diseases.

## Background

Motor neuron diseases are a group of lethal neurological disorders with different etiologies and clinical variability that have one characteristic in common: the selective degeneration of motor neurons, a small population of cells within the central nervous system with a highly specialized morphology and function [1]. To study the selective vulnerability of motor neurons in these diseases, isolated primary motor neurons, either cultured pure or in co-culture with glia or muscle cells, have been established as an important *in vitro *model. Studies on cultured spinal cord motor neurons have made important contributions to our understanding of motor neuron neurobiology, such as the identification of neurotrophic factors that regulate survival, regeneration and plasticity [2,3].

However, functional studies in primary motor neurons have been hampered by the lack of reliable and efficient transfection protocols to enable not only protein overexpression, but also gene knockdown with shRNA constructs. Currently, the only method with acceptable transfection efficiency is transduction with lentiviral vectors, which has some limitations (e.g. size of the insert), and is both time consuming and requires additional safety precautions and special equipment [4,5]. A representative example for a motor neuron disease where most *in vitro *studies have been performed in immortalized cell lines is spinal muscular atrophy (SMA). SMA is caused by mutations or gene deletions in the survival of motor neuron (*SMN1*) gene encoding the ubiquitously expressed SMN protein [6-8]. SMN has a critical housekeeping function in the correct assembly of spliceosomal small nuclear ribonucleoproteins (snRNPs) [9]. It is a major conundrum in SMA research how a reduction in levels of the ubiquitously expressed and essential SMN protein in all tissues selectively affects motor neurons. Since SMN-containing granules have been found to be actively transported in processes and growth cones of chick forebrain neurons [10] and axonal defects have been observed in SMA animal models [11,12], we and others have proposed an additional role for SMN in the axon that may contribute to the unique vulnerability of motor neurons to low levels of SMN protein [7,8,13-15]. However, expression of fluorescent-tagged SMN protein has never been used as a complementary approach to immunofluorescence in motor neurons, and SMN trafficking has never been studied in axons of live primary motor neurons before. To address these limitations of motor neuron cell culture models, we have established magnetofection as a novel tool for the reliable, rapid and efficient non-viral transfection of embryonic primary motor neurons. In the magnetofection method, DNA aggregated with superparamagnetic iron oxide nanoparticles is deposited on target cells by the application of a magnetic field and taken up via endocytosis [16].

We have carefully optimized the time course, media, and reagent concentration for primary motor neurons to routinely achieve transfection efficiencies higher than 45%. Importantly, survival and morphology of the transfected motor neurons were not altered. Magnetofection allows both efficient overexpression and knockdown of the gene of interest to study the effects of both toxic gain of function and loss of function on the differentiation and survival of primary motor neurons.

Using this method, we have shown for the first time the active transport of the survival of motor neuron (SMN) protein along the axon of live primary motor neurons.

The described method has not been used in motor neurons before and represents a powerful new tool to study the role of proteins implicated in SMA and other motor neuron diseases in the very cell type that is primarily affected by these disorders.

## Results and Discussion

### Primary motor neurons can be efficiently transfected with magnetic nanobeads

In an effort to overcome fundamental limitations of viral and non-viral gene transfer into primary cultured motor neurons, we have tested transfection with magnetic nanobeads (magnetofection), a method that has been previously used on primary hippocampal neurons [17]. Postmitotic neurons are sensitive to cytotoxicity and difficult to transfect. This is especially true for primary motor neurons, in which the efficiency of conventional transfection techniques (i.e. liposomes, electroporation) is far too low for most applications or the transfection procedures are cytotoxic, inducing morphological changes and cell death. Whereas in hippocampal neurons several transfection methods such as nucleofection [18], liposomes [19], Polyethylenimine [20] and Ca(2+)-phosphate [21] have been established, currently there is no efficient method for the transfection of primary motor neurons available.

**Figure 1:**
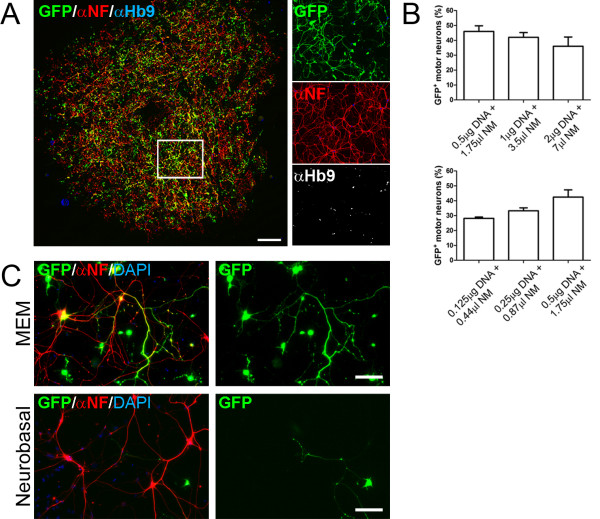
**Magnetofection of primary motor neurons is highly efficient**. Primary motor neurons (2 DIV) were transfected with pmaxGFP using magnetic nanobeads. Different amounts of DNA and beads and different media were tested to optimize transfection efficiency. Cells were fixed 2 days post transfection and stained using neurofilament (NF) and HB9 antibodies to identify motor neurons. **A**. Representative image of a motor neuron culture from an entire 15 mm diameter coverslip. **B**. GFP/HB9^+ ^cells were scored and normalized to the number of HB9^+ ^cells. Maximum efficiency of transfection (46%) was achieved using 0.5 μg DNA and 1.75 μl beads (mean and SEM, n = 3). **C**. Neurobasal medium inhibited motor neuron transfection when used during DNA/beads complex formation. MEM is shown as a positive control. Size bars: 1 mm in A, 50 μm in B.

To test the efficiency of magnetofection on primary motor neurons, we performed preliminary experiments transfecting a plasmid encoding a variant of green fluorescent protein (pmaxGFP) into 2 DIV primary motor neurons, the stage at which the motor neuron axon is still actively growing. In order to maximize transfection efficiency, we varied the total DNA amount used, ranging from 0.5 μg to 2 μg, but we kept the DNA:beads ratio constant (1:3.5). Cells were fixed 48 hours post transfection and stained for neurofilament and the motor neuron specific marker HB9. Whole 15 mm coverslips were imaged and the percentage of GFP-positive motor neurons was evaluated under different conditions (Fig. 1A). By lowering the DNA amount to 0.5 μg, we achieved transfection efficiencies higher than 45% (Fig. 1B). However, further reducing DNA levels down to 0.125 μg did not prove beneficial, decreasing the percentage of transfected cells to less than 30%. Since motor neurons are cultured in Neurobasal medium, we decided to compare it with minimal essential medium (MEM) during the DNA/beads complex formation. Surprisingly, we found that using Neurobasal medium during DNA/beads complex formation resulted in very low transfection efficiencies for motor neurons (Fig. 1C).

**Figure 2:**
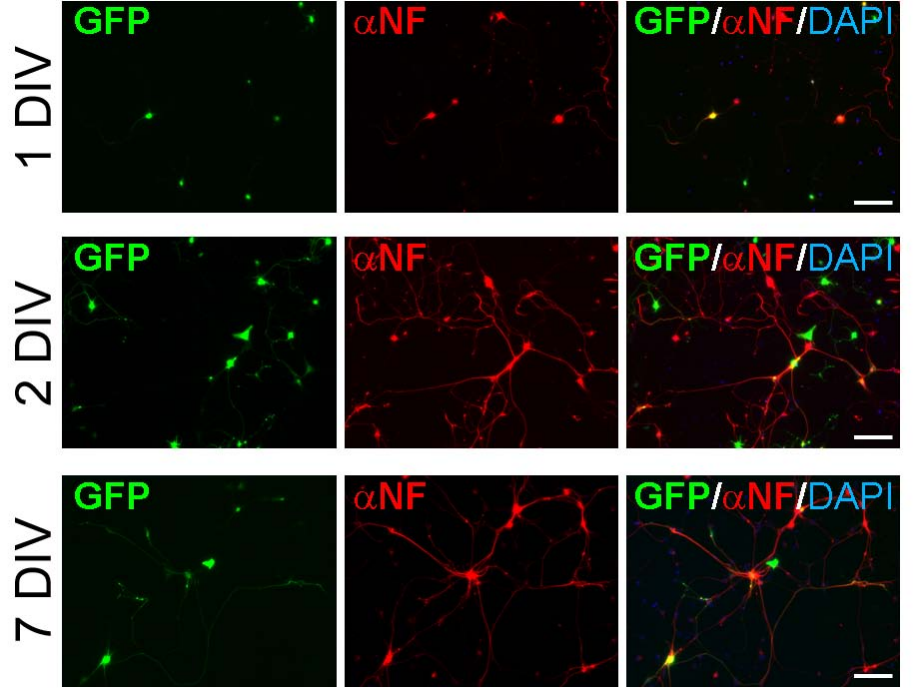
**Young and mature motor neuron cultures are efficiently transfected by magnetofection**. 1 DIV (*top*), 2 DIV (*middle*), and 7 DIV (*bottom*) motor neurons were transfected with pmaxGFP. Twenty-four hours after transfection cells were fixed and stained with neurofilament antibody to identify neurons (NF, red). DAPI was used to stain nuclei (blue). GFP-positive cells (green) are visible in all tested conditions. Size bars: 100 μm.

The ability to transfect motor neurons at different days after plating is very important for studying the effect of expressing a specific protein during the initial phase of axon outgrowth or in more mature neurons. To assess whether our method would be suitable for this application, we transfected the pmaxGFP plasmid into young (0 and 1 DIV) and more mature (7 DIV) cultures. While the magnetofection of motor neurons at the time of plating (0 DIV) resulted in high toxicity and cell death (data not shown), both 1 DIV and 7 DIV motor neurons were efficiently transfected using our protocol, although with a lower efficiency than 2 DIV cultures (Fig. 2).

Taken together, these data show that the use of paramagnetic nanobeads represents a new and highly efficient method to transfect primary cultured motor neurons at different stages of differentiation and maturation. On average, two thirds of the cells obtained from one single lumbar spinal cord were used per magnetofection. Using coverslips smaller than 15 mm would further reduce the required cell number. Importantly, this shows that our protocol works with low cell numbers and therefore can be used to perform multiple transfections of motor neurons from single mouse embryos, for example to compare the effect of gene expression in wild type and knockout motor neurons.

### Primary motor neurons show normal morphology and survival after magnetofection

In parallel to quantifying transfection efficiencies, we investigated potential direct toxic effects or cell membrane perturbations caused by the magnetic beads, that could cause changes to cell morphology and survival. Magnetic nanoparticles are made of biodegradable iron oxide with a polymer coating and have been shown in other cell types to be non-toxic and not to influence cellular functions [17,22-24]. Primary motor neurons, however, are especially sensitive to changes to the environment and may be more susceptible to cytotoxicity than other cell types.

**Figure 3:**
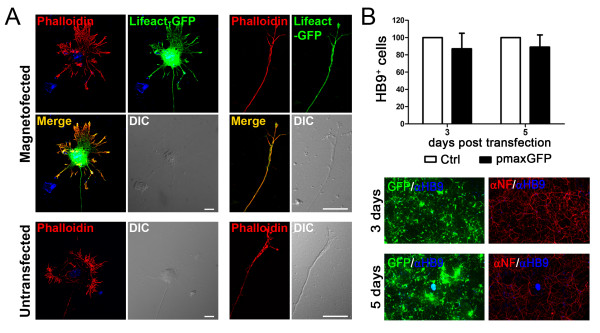
**Magnetofection does not affect motor neuron morphology and survival**. Primary motor neurons (2 DIV) were transfected with Lifeact-GFP (A) or pmaxGFP (B) and fixed 1 day (A), or 3 and 5 days (B) after transfection. **A**. Motor neuron morphology was evaluated using the F-actin probe Lifeact-GFP and compared to rhodamine phalloidin staining. Cell body, dendrites (*left*) and growth cone (*right*) did not show any difference between transfected (*top panel*) and control (*bottom*) cells. Size bar: 10 μm. **B**. Surviving motor neurons were scored based on neurofilament (NF) and HB9 staining (*left*). Representative images are shown (*bottom right*). Bars represent mean and SEM from 3 independent experiments. One way repeated measures ANOVA, p > 0.05.

In order to investigate changes in cell morphology caused by magnetofection, we transfected motor neurons with a plasmid encoding the F-actin reporter Lifeact-GFP [25]. The Lifeact peptide has been shown to bind actin filaments *in vivo *without affecting their dynamics. Cells were fixed 24 hours after transfection, and the morphology of the cell body, dendrites, and axonal growth cone was compared with untransfected cells stained with the fluorescently labeled F-actin probe rhodamine phalloidin (Fig. 3A). No obvious changes in any of the cell compartments analyzed were observed. We then investigated whether survival of motor neurons was affected by the delivery of the magnetic beads. For this purpose, cells were transfected with the pmaxGFP plasmid and fixed 3 and 5 days post transfection. When we compared the number of HB9-positive neurons in transfected and untransfected cultures, we did not observe any differences (Fig. 3B). Importantly, the percentage of GFP-positive motor neurons did not decrease over the course of 5 days (46.2 ± 6.7% and 43.13 ± 5.17% at day 3 and 5, respectively) confirming the low toxicity of the transfection method.

Taken together, these data demonstrate that motor neurons can be efficiently transfected without a negative impact on their survival and morphology, and that gene expression persists at a high level several days after transfection, establishing the magnetofection method as a valuable tool for studying motor neuron biology.

### Several constructs can be co-transfected efficiently into primary motor neurons

**Figure 4:**
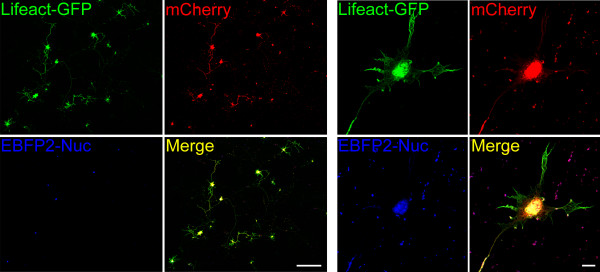
**Magnetofection allows high-efficient transfection using multiple constructs**. Primary motor neurons were transfected using three different constructs coding for the F-actin binding protein Lifeact fused to GFP, the soluble red fluorescent protein mCherry, and the nuclear localized blue fluorescent protein EBFP2-Nuc. **A**. All transfected cells expressed all three fluorescent proteins. **B**. High magnification shows the different subcellular localization of all three proteins in the same cell. Background dots in the red and blue channels are due to remaining autofluorescent beads. Size bars: 200 μm in A and 10 μm in B.

One of the main limitations of virus transduction is the difficulty to co-deliver several constructs into the same cell due to viral interference [26]. To address the possibility to overcome this limitation using our method, we transfected motor neurons with three different plasmids encoding for the soluble red fluorescent protein mCherry [27], the F-actin binding fusion protein Lifeact-GFP [25], and the nuclear-targeted blue fluorescent protein EBFP2-Nuc [28]. All transfected motor neurons showed expression of all three proteins correctly localized to different cell compartments (Fig. 4). This result demonstrates that the co-expression of at least three proteins in the same cell is feasible. Taken together our data support magnetofection as a very useful alternative to viral transduction. It also has several important advantages: it is more reproducible and less time consuming because it does not depend on cloning into transfer vectors, packaging into viral particles, and determining the lentivirus titers. Also, there is no size limit on the insert size of the plasmid and efficient co-transfection of several constructs into one cell is not affected by viral interference [26]. Additionally, unlike the delay observed with viral transduction, high expression levels can be achieved as early as one day after transfection.

### Axonal localization and bi-directional axonal transport of Smn in transfected motor neurons

As of now, the vast majority of studies on SMN function have been performed in non-neuronal cells (i.e. fibroblasts) that do not represent the cell population affected in SMA. Due to the lack of reliable transfection methods, SMN localization and trafficking using fluorescent reporters has yet to be studied in motor neurons.

**Figure 5:**
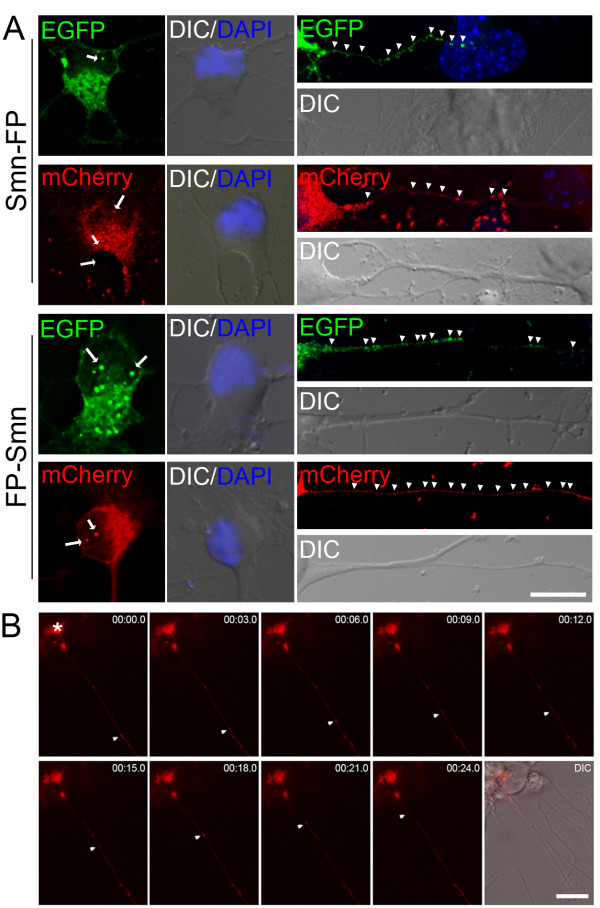
**Fluorescent protein-tagged Smn is localized and actively transported in the axons of primary motor neurons after magnetofection**. A. Full-length Smn fused to the N- (*top*) or C-terminus (*bottom*) of EGFP or mCherry fluorescent proteins (FP) was tested in order to exclude artefacts caused by the fusion partner. Compressed deconvolved 3-D image stacks show Smn-positive gems in the nucleus (arrows) and small granules localized along the axon (arrowheads). DAPI staining (blue) identifies nuclei. B. Live cell imaging of Smn-mCherry granules. Single snapshots of a representative Smn-positive granule moving anterogradely toward the growth cone (*star*) are shown. The granule moved 50.05 μm with an average speed of 1.80 μm/sec. Size bars: 10 μm in A, 15 μm in B.

For this reason, we decided to apply our novel method to the study of the localization of murine Smn in the axons of primary motor neurons. For this purpose, we used both N- and C- terminal fusions of full length Smn with EGFP and mCherry proteins. In all tested combinations, Smn protein localized to nuclear foci, called gems, and to small granules in the cytoplasm, and along the dendrites and axons of primary motor neurons (Fig. 5A), well in agreement with the localization of the endogenous Smn protein [8,13,15].

Since our results have shown that magnetofection has no apparent toxic effect on motor neurons, we attempted to use this method for studying the axonal transport of Smn. Performing successful live-cell imaging experiments demands that the cells remain in a healthy state and that cell morphology and function are preserved during examination under the fluorescence microscope. To study trafficking of Smn-containing granules in the axon of living motor neurons, we transfected an expression construct for Smn tagged with mCherry into primary motor neurons. In order to increase the chances to observe moving granules, cells were starved with medium lacking trophic factors before adding complete glia-conditioned imaging medium supplemented with 100 μM of the cell-permeable cAMP analogue 8-Bromo-cAMP, a paradigm known to stimulate β-actin mRNA granule transport [29]. Using time-lapse video microscopy, we could show for the first time that Smn is actively transported in axons of primary motor neurons, both retro- and anterogradely (Fig. 5B and additional file 1). Smn-containing granules moved at a speed of 2.32 ± 0.7 μm/sec. No differences were observed between particles moving anterogradely toward the growth cone (2.21 ± 0.37 μm/sec; n = 5) or retrogradely toward the cell body (2.46 ± 1.02 μm/sec; n = 4). These results show that magnetofection can meet the technical challenges presented by live cell imaging and allow for analysis of axonal protein transport in primary motor neurons.

### Magnetofection can be used to silence gene expression

**Figure 6:**
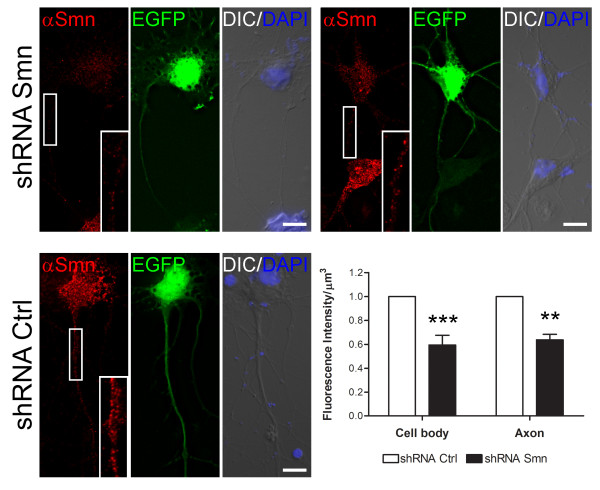
**Smn knockdown in primary cultured motor neurons after magnetofection with an shRNA construct**. Primary motor neurons were transfected with an Smn-specific (*top*) or control shRNA plasmid (*bottom*) for 5 days. Transfected cells were identified by EGFP expression (green). Anti-Smn antibody staining (red) was used to evaluate Smn protein levels. Fluorescence intensity was quantified in the cell body and axon using the Imaris software and normalized to the axon volume. Smn protein levels were reduced in both cell body and axon when the Smn-specific shRNA was used, in comparison to the control shRNA construct. Bars represent mean and SEM. One way ANOVA, n = 3; ***p < 0.001, **p < 0.01. Size bar: 10 μm.

One of most powerful approaches to study the function of a protein is to knockdown its expression with siRNA technology. The efficiency of gene transfer using our protocol prompted us to investigate the possibility not only to overexpress a gene product, but also to silence its expression using a vector-based approach. For this purpose, we transfected motor neurons either with an shRNA vector targeting Smn mRNA or a non-silencing control. Cells were fixed after 5 days, and stained for endogenous Smn protein. Transfected cells expressing the shRNA construct, identified by GFP expression, showed a significant reduction in endogenous Smn protein levels in cell bodies and axons by 40.6% and 36.2%, respectively, when compared to the non-silencing control shRNA vector (Fig. 6). Our results show that expression from transfected plasmids was strongly visible at 12 h after transfection and persisted at a high level several days after transfection allowing gene silencing by shRNA technology. Importantly, we can show reduced levels of Smn not only in the cell body, but also in the axonal compartment, allowing investigations of the axonal function of specific genes and proteins in primary motor neurons. Our results demonstrate that magnetofection is a useful tool to study the role of specific proteins in axons of primary motor neurons.

## Conclusions

In this study we have established magnetofection as a novel method for the transfection of primary motor neurons that is efficient, robust and non-toxic. It is cost-effective, easy to perform, and does not require specialized equipment. Our optimized protocol allows for efficient transfection of motor neurons during different stages of maturation, high levels of expression over several days, and the co-expression of at least three proteins in one cell.

To test the application of this promising new method, we have for the first time overexpressed and knocked-down the SMA disease protein Smn in the cell body and axon of motor neurons. We have also shown for the first time the active transport of Smn-containing granules along motor neuron axons anterogradely and retrogradely with a speed consistent with fast axonal transport.

We anticipate that this efficient transfection method for primary motor neurons and its applications described in this study will also have more general applications in neurodegeneration research. We expect that this approach will facilitate the research on pathways regulating motor neuron development and survival as well as molecular mechanisms underlying motor neuron disease.

## Methods

### Embryonic primary motor neuron culture

C57BL6 timed pregnant mice (Charles Rivers) were sacrificed following the protocol approved by the Institutional Animal Care and Use Committee (IACUC) at Emory University. Primary motor neurons from E13.5 mouse embryos were isolated and cultured essentially as described [30,31], but the magnetic column step was omitted, and 6% Optiprep (Sigma) was used for gradient centrifugation. Glia cells obtained from embryonal spinal cords after motor neuron isolation were grown in MEM, 10%FBS, 10 mM Hepes, 33 mM glucose. Secondary glia cells were incubated with motor neuron culture medium (Neurobasal, 0.5 mM Glutamax, 2% B27; Invitrogen) for one day to obtain glia-conditioned media. Motor neurons were plated on 15 mm coverslips (Assistant cover glasses; Carolina) coated with 0.5 mg/ml poly-ornithine (MW 30-70 KDa; Sigma) and 0.5 mg/ml laminin (Invitrogen), and cultured in glia-conditioned Neurobasal/B27 medium supplemented with 2% horse serum (Sigma), and 10 ng/ml each BDNF, CNTF, and GDNF (Peprotech).

### Magnetofection of primary motor neurons

Motor neurons (2 DIV) were transfected using paramagnetic nanobeads (NeuroMag, Oz Biosciences) as follows: complete medium was replaced 1 hour prior to magnetofection with serum-free glia-conditioned Neurobasal/B27 medium. Plasmid DNA (0.5 μg) was incubated with 1.75 μl NeuroMag beads in 100 μl minimal essential medium (MEM) for 15 minutes, and then added drop-wise to the cultures. Cells were incubated on top of a magnetic plate (Oz Biosciences) for 15 minutes and complete medium was restored after 1 hour.

### Constructs

mCherry [27], Lifeact-GFP [25] and pEBFP2-Nuc [28] were kindly provided by Dr. Roger Tsien (UCSD, USA), Dr. Wedlich-Soldner (MPI of Biochemistry, Germany), and Dr. Campbell (University of Alberta, Canada; Addgene plasmid #14893). The pmaxGFP vector was from Lonza. The SMN (V2LMM_6035) and control (RHS4346) shRNA vectors were from Open Biosystems. Green (EGFP) or red (mCherry) fluorescent proteins were fused N- or C-terminal to Smn. A flexible liker [(SGGG)_3_] was inserted between the fusion partners to facilitate correct protein folding. A point mutation was introduced (A206K) to generate monomeric forms of GFP as described [32].

### Cell staining and imaging

Magnetofected motor neurons were fixed for 15 minutes with 4% paraformaldehyde 24 hours to 5 days after transfection, as indicated. Anti- neurofilament (1:500; 2H3, DHSB), HB9 (1:5000; Abcam), and SMN (1:500; BD) antibodies were incubated overnight at 4°C. Rhodamine-conjugated phalloidin (1:500; Invitrogen), and Cy3-, Cy2- or Cy5-conjugated secondary antibodies were incubated for 1 hour at room temperature. Cells were imaged using a widefield microscope (TE300, Nikon) equipped with a high resolution CCD camera (Quantix, Photometrics). Image stacks were deconvolved (Autodeblur, Media Cybernetics) and analyzed using the Imaris software (Bitplane). Entire 15 mm coverslips were scanned using a motorized confocal microscope (LSM 510, Zeiss). The 2H3 antibody developed by Dr. Jessell was obtained from the Developmental Studies Hybridoma Bank (DSHB) developed under the auspices of the NICHD and maintained by The University of Iowa, Department of Biology, Iowa City, IA 52242.

### Life cell imaging

Motor neurons were plated on poly-ornithine/laminin coated Delta T culture dishes (Bioptechs). Twenty-four hours after transfection, cells were starved in plain Neurobasal medium for 30 minutes and then stimulated for 15 minutes with 100 μM 8-Bromo-cAMP (Calbiochem) in glia-conditioned imaging medium (Hibernate E low fluorescence, Brain Bits) supplemented with 2% B27 and 2% horse serum. Movies were acquired using a widefield epifluorescence microscope (TE2000, Nikon) with a high speed cooled CCD camera (Cascade 512 b, Photometrics).

## Competing interests

The authors declare that they have no competing interests.

## Authors' contributions

CF performed experiments, data analyses and participated in the design of the study. WR conceived of the study, designed and assisted with experiments, and co-wrote the manuscript with CF. GB participated in the design and coordination of experiments and helped to draft the manuscript. All authors read and approved the final manuscript.

## Supplementary Material

Additional file 1 Life cell imaging of an Smn-mCherry granule in transfected motor neurons. Click here for file
